# Outbreaks of serotype 4 fowl adenovirus with novel genotype, China

**DOI:** 10.1038/emi.2016.50

**Published:** 2016-05-25

**Authors:** Jianqiang Ye, Guangchen Liang, Jianjun Zhang, Weikang Wang, Na Song, Ping Wang, Wenlv Zheng, Quan Xie, Hongxia Shao, Zhimin Wan, Chengming Wang, Hongjun Chen, Wei Gao, Aijian Qin

**Affiliations:** 1 Ministry of Education Key Laboratory for Avian Preventive Medicine, and Key Laboratory of Jiangsu Preventive Veterinary Medicine, Yangzhou University, Yangzhou, Jiangsu 225009, China; 2 Jiangsu Co-innovation Center for Prevention and Control of Important Animal Infectious Diseases and Zoonoses, Yangzhou, Jiangsu 225009, China; 3 Sinopharm Yangzhou VAC Biological Engineering Co., LTD, Yangzhou, Jiangsu 225009, China; 4 Shanghai Veterinary Research Institute, Chinese Academy of Agricultural Sciences, Shanghai 200241, China

**Dear Editor,**

Fowl adenovirus (FAdV) belongs to the genus *Aviadenoviru*s and has been clustered into five species (A–E) with 12 serotypes.^1^ Although this virus has spread globally, FAdV infection typically causes only subclinical symptoms, and severe FAdV infection is associated with inclusion body hepatitis (IBH), hydropericardium syndrome (HPS) and gizzard erosion and ulceration.^1, 2, 3, 4, 5, 6, 7, 8^ Notably, severe FAdV cases with IBH and HPS in chicken flocks have been observed in China since 2013.^9^ These outbreaks typically occurred in 3–5-week-old broiler flocks, as well as in 10–20-week-old layer flocks. In contrast to mild disease caused by previous FAdV outbreaks, the outbreaks of FAdV in 2015 in China caused a huge economic loss in the poultry industry. Surprisingly, to date, little is known regarding the molecular characteristics or serotypes of these potentially devastating FAdVs that remain in circulation in China.

To identify which serotypes or variants of FAdV currently circulate in chicken flocks in China, we isolated FAdV from five provinces (Jiangsu, Shangdong, Anhui, Henan and Jiangxi) in 2015 for sequence analyses. For viral isolation, approximately 150 chickens with IBH or HPS were collected from the five provinces, and chicken liver homogenates were inoculated into embryonated chicken eggs. The isolates were identified as FAdV-positive by polymerase chain reaction (PCR) with primers (forward: 5′-CAA CTA CAT CGG GTT CAG GGA TAA CTT C-3′, reverse: 5′-CCA GTT TCT GTG GTG GTT GAA GGG GTT-3′), which are highly conserved in all the five species of FAdV, and specifically amplify a 766-bp fragment of the hexon gene (from position 852 to 1518 based on the hexon gene of strain ON1). The PCRs were performed with the following protocol: 94 °C for 5 min followed by 30 cycles of 94 °C for 30 s, 55 °C for 30 s, 72 °C for 30 s and 72 °C for 10 min. Next, the PCR products were sequenced by Sanger sequencing. In the end, nine FAdVs were isolated from the five provinces in 2015 and named JSCZ15, JSSQ15, JSTZ15, SD15, SDWF15, AHFY15, AHHF15, JX15 and HNLY15. Isolates JSCZ15, JSSQ15 and JSTZ15 were from Jiangsu; isolates SD15 and SDWF15 were from Shangdong; isolates AHFY15 and AHHF15 were from Anhui; and isolates JX15 and HNLY15 were from Jiangxi and Henan, respectively. Among these isolates, only JSSQ15 (11.11%) was identified as serotype 8 FAdV, while the other eight isolates (88.89%) were identified as serotype 4 FAdV. The PCR product of JSSQ15 showed 100% homology with that of serotype 8 (FAdV-E), whereas the PCR products from other eight isolates had 100% identity to that of serotype 4 (isolate ON1). This finding demonstrates that serotype 4 FAdV is epidemic in recent chicken flocks in China.

To investigate the potential molecular variations in the FAdVs isolated in this study, the genomic DNA of isolates JSCZ15, SD15, SDWF15 and AHFY15 were first extracted and then sequenced as previously described.^9^ Surprisingly, in comparison with the serotype 4 FAdV (JSJ13) isolated in 2013 in China, deletions with different sizes in ORF29 were found in the 2015 FAdV isolates. Sixty-six-nucleotide deletions were present in the genome of SD15, whereas 33-nt deletions were present in JSCZ15, SDWF15 and AHFY15 (Figure 1). Next, a region covering ORF29 of isolates JSCZ15, JSTZ15, AHHF15, JX15 and HNLY15, and another 2013 isolate JH2013 was amplified and sequenced. Interestingly, the 2013 isolate JH2013, similar to JSJ13, did not carry a deletion in ORF29, whereas the 2015 isolates JSCZ15, JSTZ15, JX15, HNLY15 and AHFY15 all had a 33-nt deletion in ORF29. Notably, additional whole genome sequence analyses revealed a deletion of 1961 nt from position 35413 to 37373 in four Chinese serotype 4 FAdVs compared with other serotype 4 isolates (KR-5, ON1 and MX-SHP90 from Austria, Canada and Mexico, respectively) available in NCBI. This deletion covered ORF19 (coding lipase) and ORF48 based on the sequence of the prototype serotype 4 isolate ON1. By contrast, the fiber1, fiber2 and hexon proteins associated with the antigenicity for these Chinese FAdVs showed 94.4%–94.7%, 93.3%–94.5% and 98.4%–98.6% similarity, respectively, to those from KR-5, ON1 and MX-SHP90, indicating potential antigenic variation. However, these proteins among the Chinese FAdVs had 99.8%–100% identity to each other.

Our data demonstrated that serotype 4 FAdV with a novel genotype was epidemic in China. These novel Chinese serotype 4 FAdVs from chicken flocks with IBH and HPS outbreaks carry a deletion of ORF19. Another 2015 Chinese serotype 4 FAdV HB1510 isoalted by Li *et al.* and made available in NCBI also shows the ORF19 deletion. Although the role of this deletion is unknown, a recent study by Pedro *et al.* indicated that serotype 4 FAdV isolates with truncated ORF19 showed higher virulence than isolates with the full ORF19.^10^ This finding suggests that Chinese serotype 4 FAdVs with deletions of ORF19 may be highly pathogenic. Our recent infection studies in chickens with isolates JSCZ15, SD15 and SDWF15 resulted in 80–100% mortality, confirming the high virulence of these FAdVs. However, the exact role of the deletion of ORF19 in these FAdVs still needs to be elucidated. Our data also revealed that 2015 Chinese serotype 4 FAdVs had 33-nt or 66-nt deletions in ORF29 in comparison with 2013 Chinese serotype 4 FAdV (Figure 1). A 33-nt deletion is also found in isolate HB1510. This finding highlighted the host adaptation of these serotype 4 FAdVs in recent chicken flocks in China. Further analysis revealed that isolates MX-SHP90 and KR-5 from other continents also carried a deletion of 33 nt in ORF29, and a 66-nt deletion was observed in isolates ON1. However, little is known regarding the roles of these deletions or host adaptation in the pathogenicity of these novel Chinese serotype 4 FAdVs. Future studies should investigate the large geographic distribution of these novel serotype 4 FAdVs, monitor their variants, identify their virulence determinants and develop efficient strategies against these novel serotype 4 FAdVs.

## Figures and Tables

**Figure 1:**
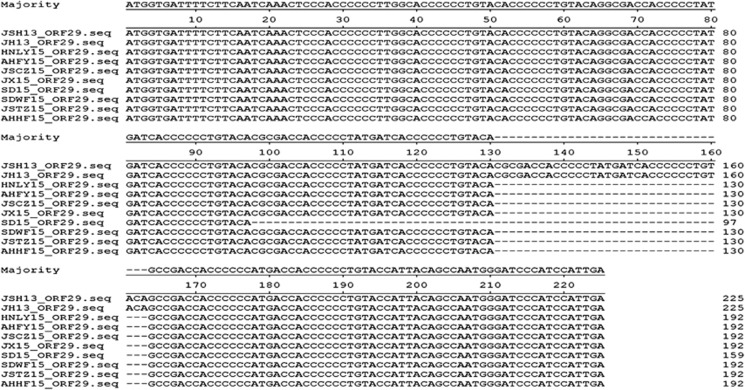
Alignment of ORF29 from recent Chinese serotype 4 fowl adenovirus. The ORF29 sequences of these Chinese serotype 4 fowl adenovirus, including nine 2015 isolates from this study (JSCZ15, JSSQ15, JSTZ15, SD15, SDWF15, AHFY15, AHHF15, JX15 and HNLY15) and two 2013 isolates (JSJ13 and JH13), were aligned using DNAStar.

## References

[bib1] Harrach B, Benko M, Both GW et al. Family Adenoviridae. In: King AMQ, Adams MJ, Carstens EB, Lefkowitz EJ (eds). Virus taxonomy. 9th ed. San Diego: Elsevier Academic Press, 2012, 125–141.

[bib2] Niczyporuk JS. Phylogenetic and geographic analysis of fowl adenovirus field strains isolated from poultry in Poland. Arch Virol 2016; 161: 33–42. 26446890 10.1007/s00705-015-2635-4

[bib3] Okuda Y, Ono M, Shibata I, Sato S. Pathogenicity of serotype 8 fowl adenovirus isolated from gizzard erosions of slaughtered broiler chickens. J Vet Med Sci 2004; 66: 1561–1566. 15644608 10.1292/jvms.66.1561

[bib4] Mittal D, Jindal N, Tiwari AK, Khokhar RS. Characterization of fowl adenoviruses associated with hydropericardium syndrome and inclusion body hepatitis in broiler chickens. Virusdisease 2014; 25: 114–119. 24426318 10.1007/s13337-013-0183-7 PMC3889237

[bib5] Wells RJ, Harrigan K. A fatal adenovirus infection of broiler chickens: inclusion body hepatitis. Vet Rec 1974; 94: 481–482. 4211755 10.1136/vr.94.21.481

[bib6] Choi KS, Kye SJ, Kim JY et al. Epidemiological investigation of outbreaks of fowl adenovirus infection in commercial chickens in Korea. Poult Sci 2012; 91: 2502–2506. 22991534 10.3382/ps.2012-02296

[bib7] Asthana M, Chandra R, Kumar R. Hydropericardium syndrome: current state and future developments. Arch Virol 2013; 158: 921–931. 23242777 10.1007/s00705-012-1570-x

[bib8] Grgic H, Krell PJ, Nagy E. Comparison of fiber gene sequences of inclusion body hepatitis (IBH) and non-IBH strains of serotype 8 and 11 fowl adenoviruses. Virus Genes 2014; 48: 74–80. 24142408 10.1007/s11262-013-0995-y

[bib9] Zhao J, Zhong Q, Zhao Y, Hu YX, Zhang GZ. Pathogenicity and complete genome characterization of fowl adenoviruses isolated from chickens associated with inclusion body hepatitis and hydropericardium syndrome in China. PloS One 2015; 10: e0133073. 26167857 10.1371/journal.pone.0133073 PMC4500579

[bib10] Vera-Hernandez PF, Morales-Garzon A, Cortes-Espinosa DV et al. Clinicopathological characterization and genomic sequence differences observed in a highly virulent fowl aviadenovirus serotype 4. Avian Pathol 2015; 27: 1–32. 10.1080/03079457.2015.1125443 26610321

